# Pregnancy Trimester-Specific Exposure to Ambient Air Pollution and Child Respiratory Health Outcomes in the First 2 Years of Life: Effect Modification by Maternal Pre-Pregnancy BMI

**DOI:** 10.3390/ijerph15050996

**Published:** 2018-05-15

**Authors:** Shu-E Soh, Anne Goh, Oon Hoe Teoh, Keith M. Godfrey, Peter D. Gluckman, Lynette Pei-Chi Shek, Yap-Seng Chong

**Affiliations:** 1 Singapore Institute for Clinical Sciences, Agency for Science, Technology and Research, Singapore 117549, Singapore; pd.gluckman@auckland.ac.nz (P.D.G.); lynette_shek@nuhs.edu.sg (L.P.-C.S.); obgcys@nus.edu.sg (Y.-S.C.); 2 Department of Paediatrics, Yong Loo Lin School of Medicine, National University of Singapore, Singapore 119228, Singapore; 3 Department of Paediatrics, KK Women’s and Children’s Hospital, Singapore 229899, Singapore; anne.goh.e.n@singhealth.com.sg (A.G.); teoh.oon.hoe@singhealth.com.sg (O.H.T.); 4 MRC Lifecourse Epidemiology Unit and NIHR Southampton Biomedical Research Centre, University of Southampton and University Hospital Southampton NHS Foundation Trust, Southampton SO16 6YD, UK; kmg@mrc.soton.ac.uk; 5 Liggins Institute, University of Auckland, Auckland 1023, New Zealand; 6 Department of Obstetrics and Gynaecology, Yong Loo Lin School of Medicine, National University of Singapore, Singapore 119074, Singapore

**Keywords:** pregnancy, trimester, respiratory, air pollution, pre-pregnancy body mass index (BMI)

## Abstract

Prenatal exposure to air pollution is associated with childhood respiratory health; however, no previous studies have examined maternal pre-pregnancy body mass index (BMI) as a potential effect modifier. We investigated whether maternal pre-pregnancy BMI modified the association of trimester-specific air pollution divided into quartiles of exposure (Q1–4) on respiratory health in the Growing Up in Singapore towards healthy Outcomes (GUSTO) study (*n* = 953) in 2-year-old children. For episodes of wheezing, children of overweight/obese mothers and who were exposed to particulate matter less than 2.5 μm (PM_2.5_) in the first trimester had an adjusted incidence rate ratio (IRR) (95% confidence interval (CI)) of 1.85 (1.23–2.78), 1.76 (1.08–2.85) and 1.90 (1.10–3.27) in quartile (Q) 2–4, with reference to Q1. This association is seen in the second trimester for bronchiolitis/bronchitis. The risk of ear infection in the first year of life was associated with exposure to PM_2.5_ in the first trimester with adjusted Odds Ratio (adjOR) (95% CI) = 7.64 (1.18–49.37), 11.37 (1.47–87.97) and 8.26 (1.13–60.29) for Q2–4, and similarly in the second year with adjOR (95% CI) = 3.28 (1.00–10.73) and 4.15 (1.05–16.36) for Q2–3. Prenatal exposure to air pollution has an enhanced impact on childhood respiratory health, and differs according to maternal pre-pregnancy BMI.

## 1. Introduction

The World Health Organization (WHO) recently announced that environmental risk factors such as air pollution, water contamination and inadequate hygiene contributed annually to nearly 1.7 million child deaths [1]. Approximately 600,000 pre-school aged children died annually due to polluted air [2]. In the most recent report by the Global Burden of Disease (GBD) project, exposure to fine particulate matters (diameter less than 2.5 μm, PM_2.5_) was ranked 5th worldwide as a risk factor for death [3]. Alarmingly, more than 90% of the global population resided in areas with polluted air and South-East Asia was among the regions with the highest concentration of PM_2.5_ in 2015 [3]. Air pollution is not an issue to be overlooked. Maternal exposure to common air pollutants such as sulphur dioxide (SO_2_), nitrogen dioxide (NO_2_), particulate matter (diameter less than 10 μm, PM_10_), PM_2.5_, carbon monoxide (CO) and ozone (O_3_) before conception as well as during gestation has been associated with the incidence of many adverse health outcomes in offspring. In particular, exposure to PM_10_ or PM_2.5_ has been extensively studied, due to their ability to travel deep into the inhaler’s lungs and cause serious health issues [4]. The sustained influence of the early environment on health over the lifespan forms the basis of the “developmental origins of health and disease (DOHaD)” paradigm [5]. This suggests that environmental conditions in early life affect the development of the offspring through effects on the mechanisms of developmental plasticity, including epigenetic processes, that in some contexts affects later vulnerability for disease [6].

A study group in China found that exposure to air pollutants such as PM_10_, NO_2_ and SO_2_ during the preconception, perinatal and postnatal periods has been associated with high prevalence of early childhood eczema, asthma and allergic rhinitis, with the strongest association between NO_2_ and eczema seen during first trimester exposure [7,8]. They also reported that young children were at increased risk of otitis media when exposed to both outdoor and indoor air pollutants [9].

The lung functions in newborns were significantly affected when mothers were exposed to air pollution during gestation [10]. Increased minute ventilation was observed in newborns with elevated intrauterine PM_10_ exposure [10]. Similarly, reduced lung function was reported in preschoolers born to mothers who were exposed to higher concentrations of benzene and NO_2_ during gestation [11]. However, a moderate level of NO_2_ exposure during gestation was shown to have an insignificant effect on the respiratory health of the offspring [12].

The air quality in Singapore has generally been satisfactory, with occasional negative influence from forest fires due to the slash-and-burn agricultural practices of neighboring countries. According to the data published by the Ministry of the Environment and Water Resources (MEWR) Singapore, during the study period of 2006 to 2016, the pollutant standards index (PSI) was categorized as good and moderate on most of the days (>94% of days per year), except for 2 separate years [13]. Singapore had the worst air condition in 2013, with 3-h PSI peaked at 401 [14] and 2015, when 1-h PM_2.5_ peaked at 471 [15]. In addition to that, the air quality can deteriorate during the Hungry Ghost festival, which is celebrated by most Chinese in Singapore [16]. During the festival, large quantities of joss paper and incense are burnt openly. Moreover, due to Singapore’s limited resources for renewable energy, power generation contributed to a considerable amount of carbon emission [17].

Maternal pre-pregnancy body mass index (BMI) has been found to be associated with offspring’s respiratory health later in life. Positive correlation has been reported for illnesses such as wheezing and asthma in children up to the first 8 years of life [18,19,20,21,22]. However, to the best of our knowledge, there has been no study on the potential synergistic effects of air pollution exposure and maternal pre-pregnancy BMI starting in utero on children’s respiratory health. Furthermore, the effect of prenatal air pollution in Singapore has not been examined although there are limited studies on direct exposure effects 1 [23,24,25].

Hence, we aim to investigate the association between mothers exposed to air pollutants during trimesters of pregnancy and the respiratory health issues faced by the offspring at 2 years of age, as some respiratory health conditions such as bronchiolitis typically occurs during the first 2 years of life.

## 2. Materials and Methods

### 2.1. Study Population

The study population for this investigation was drawn from the Growing Up in Singapore towards healthy Outcomes (GUSTO) study. It is a longitudinal birth cohort study in Singapore, focusing on the developmental origins of metabolic diseases and neurodevelopment. The recruitment took place at two sites, the KK Women’s and Children’s Hospital (KKH) and the National University Hospital (NUH) in Singapore, between June 2009 and September 2010. The 1237 recruited women were at least 18 years old, attending antenatal care at either of the hospitals and were Singapore citizens or Singapore permanent residents with homogenous parental ethnic background (Chinese, Malay or Indian). Details of the study have been discussed elsewhere [26]. The study received approvals from Institutional Review Board (IRB) (reference CIRB 2009/280/D and DSRB D/09/021) and written consents from all participants. Demographic data, including maternal age, ethnicity, and educational attainment, were obtained from interviewer-administered questionnaires at the time of enrolment. Pre-pregnancy BMI, history of maternal atopy, maternal smoke exposure and environmental tobacco smoke (ETS) exposure during pregnancy were recorded. Maternal atopy is defined as positive if the mother ever had eczema, asthma or allergic rhinitis. After delivery, all routinely recorded birth data, including parity, gender and birth weight were abstracted from the hospitals’ medical case-notes. Gestational age at birth was calculated from ultrasound scan-based estimated date of delivery.

### 2.2. Environmental Pollutant Exposures

The environmental air pollutant data was provided by the National Environment Agency (NEA) of Singapore. The exposure measures provided were the daily 24-h nation-wide average pollutant standards index (PSI), and fine particulate matter with a diameter of less than 2.5 µm (PM_2.5_) concentration in µg/m^3^. PSI, which is an indicator of air quality developed by the United States Environmental Protection Agency (USEPA), reflects five pollutants in the ambient air, namely particulate matter witokeh a diameter of less than 10 µm (PM_10_), sulphur dioxide (SO_2_), nitrogen dioxide (NO_2_), carbon monoxide (CO) and ozone (O_3_). The PSI is reported on a scale of 0 to 500, with 0–50 considered good, 51–100 moderate, 101–200 unhealthy, 201–300 very unhealthy and above 300 as hazardous. PM_2.5_ was obtained as an average measured from eight stations with representation from urban stations—P03 Environment Building, P20 Bishan ITE, P28 Temasek Polytechnic, industrial stations—P17 Stagmont Camp, P31 Pandan Reservoir, suburban station—P24 Yishun ITE and roadside stations—P15 Ngee Ann Polytechnic and P32 Chin Swee. Data was extracted between 1 January 2009 and 31 December 2013.

### 2.3. Child Health Outcomes

Interviewer-administered questionnaires were conducted by trained research personnel at 3, 6, 9, 12, 15 18, and 24 months with the child’s main caregiver. The child’s health outcomes were recorded as positive if the participant provided an affirmative answer to the questions as stated in Table 1.

### 2.4. Statistical Analysis

Population characteristics for mothers and children with or without respiratory health conditions were compared using the t-test for continuous variables such as maternal age, gestational age and birth weight, while chi-squared tests were applied for categorical variables.

In order to examine trimester specific effects, we defined the days of the pregnancy according to the dating ultrasound scan’s gestational age and expected date of delivery (EDD) to divide the entire pregnancy into three trimesters. The first trimester was defined as day 1 to day 90 of pregnancy, the second trimester as day 91 to day 180, and the third trimester as day 181 to delivery. Exposure to daily 24-h nationwide average PM_2.5_ and PSI were then back extrapolated to the specific dates of each pregnancy.

We employed multivariable Poisson regression for episodes of wheezing and bronchiolitis/bronchitis ascertained from birth to 2 years of age; results are presented as incidence rate ratios (IRRs) and 95% confidence intervals (CI). The air pollutants (PM_2.5_ and PSI) were categorized into quartiles with the lowest quartile (<25 percentile) treated as a reference group. In model 1, adjustments were made for covariates of clinical relevance and classical risk factors including maternal age at delivery, ethnicity (Chinese: reference, Malay, Indians), parity (nullipara: reference vs. multipara), maternal education (university: reference vs no education/primary/secondary and post-secondary/pre-university), history of presence of maternal atopy, maternal smoke exposure during pregnancy and ETS, gestational age, baby gender (boys: reference vs. girls), birth weight. Model 2 controlled for effect of the air pollutant in other trimesters and model 3 was further performed to adjust for child postnatal smoke exposure and postnatal PM_2.5_ and PSI exposure.

Multiple logistic regression models were performed to evaluate the associations between air pollutants during pregnancy and any diagnosis of pneumonia, rhinitis, prolonged cough and ear infection in the offspring from birth to age 1 year and during the second year of life. The models were adjusted for potential confounding variables as described above. Results are presented for adjusted models as odds ratio (OR) with 95% CI.

Potential effect modification by pre-pregnancy BMI was assessed by including multiplicative interaction terms with the risk factor scores to the fully-adjusted model. Stratified analyses were then performed in participants with a BMI of <23 and ≥23, as the Asian cut-point for overweight/obese.

All statistical analyses were performed with IBM SPSS version 24.0 (IBM SPSS Statistics, Armonk, NY, USA).

## 3. Results

Out of the 1255 recruited women, 1190 of them delivered singleton infants and 953 children completed the 2-year visit. Table 2 shows the demographics and incidence of respiratory conditions, including wheezing (29.2%), bronchiolitis/bronchitis (13.7%), pneumonia (1.8%), prolonged cough (15.1%), rhinitis (44.5%) and ear infection (5.1%) in children. We observed that only maternal atopy (*p* = 0.005) was significantly associated with the presence of any respiratory conditions listed above. Maternal age at delivery, ethnicity, educational attainment, parity, maternal smoking during pregnancy and ETS, maternal pre-pregnancy BMI, gestational age, gender, birth weight and passive smoke exposure were not significantly associated with a child’s respiratory health.

The air pollution exposure levels during the entire pregnancy and across the three trimesters are summarized in Table 3. The average exposures to PM_2.5_ and PSI were 17.92 (1.31) and 35.15 (1.01), respectively. Levels in the first and second trimesters were similar but the average PM_2.5_ and PSI values in the third trimester were significantly lower than those in the first trimester (*p* < 0.001) due to 7.9% of the participants having preterm deliveries before 37 weeks.

Table 4 shows the adjusted incidence rate ratio and 95% confidence intervals for the association of PM_2.5_ and PSI divided into quartiles during each trimester of pregnancy and episodes of wheezing by 2 years of age. A total of 29.2% of toddlers had at least one episode of wheezing. The median episodes (IQR) of wheezing were 2 (1, 3), with one child having a maximum of 19 episodes in the first 2 years of life. In model 1, we found that exposure to PM_2.5_ and PSI in the first and third trimester was associated with increased episodes of wheezing.

Across the trimesters, the pollutants were weakly or moderately correlated with each other (e.g., PM_2.5_ in first trimester with PM_2.5_ in second trimester), ranging from −0.672 to 0.268. PM_2.5_ and PSI were, however, strongly correlated within each trimester, with a correlation of 0.909 to 0.928. Therefore model 2 further adjusted for the effects of PM_2.5_ or PSI in other trimesters. The effect of PM_2.5_ in the same trimester was, however, not adjusted for if the main analysis was on PSI, and vice versa. In model 2, we found that the association of episodes of wheezing and PM_2.5_ was consistently significant with adjusted IRR (95% CI) = 1.56 (1.18–2.06) in the highest quartile (Q4) in the first trimester and 1.56 (1.07–2.28) in the third trimester. The association with PM_2.5_ in the second trimester was no longer significant after multi-trimester adjustment. Likewise, PSI remained significantly associated in the first and third trimester. 

In model 3, we proceeded to adjust for postnatal factors, including ETS and ambient air pollution (PM_2.5_ or PSI) from birth to 2 years of age. The association of PM_2.5_ with episodes of wheezing was significant in the first and third trimester with IRR (95%) = 1.60 (1.08–2.38) in Q4 of the third trimester. The effects of PSI can be seen in the first trimester with IRR (95%) = 1.47 (1.14–1.90) and 1.68 (1.20–2.34) in Q2 and Q4 respectively.

In addition, we conducted stratified analyses to assess effect modification by maternal pre-pregnancy BMI as the interaction term *p*-value was <0.05. Stratified regressions found that those born to mothers with pre-pregnancy BMI over or equal to 23 (i.e., overweight/obese in Asian context) were at an increased risk of developing more episodes of wheezing. In model 3, these children whose mothers were exposed to Q2, 3, and 4 of PM_2.5_ in the first trimester had an IRR (95% CI) of 1.85 (1.23–2.78), 1.76 (1.08–2.85) and 1.90 (1.10–3.27) respectively, indicating that PM_2.5_ exposure in the first trimester may increase the episodes of wheezing in toddlers.

Bronchiolitis/bronchitis occurred in 13.7% of the children, with a median (IQR) of 1 (1, 2) episodes. Table 5 shows the adjusted IRR and 95% CI for the association of PM_2.5_ and PSI in quartiles during each trimester of pregnancy and episodes of bronchiolitis/bronchitis by 2 years of age. In model 2 with multi-trimester adjustment, we found that the associations of episodes of bronchiolitis with PM_2.5_ and PSI were significant with adjusted IRR (95% CI) = 2.29 (1.34–3.89), 3.71 (2.05–6.70), 2.37 (1.38–4.09) in Q2–4 for PM_2.5_ in the second trimester. This effect was amplified when only the overweight and obese mothers were taken into account. As a comparison, the adjusted IRRs (95% CI) were now doubled or tripled to 5.19 (2.20–12.24), 11.06 (4.12–29.65), 4.84 (1.90–12.36), respectively, for PM_2.5_ Q2–4. The same pattern was observed in the second trimester when further adjustments were made for postnatal factors in model 3.

We further examined effects of air pollution on the occurrence of other respiratory health outcomes. Table 6 shows the adjusted odds ratio and 95% CI for the association of ambient air pollution (PM_2.5_ and PSI in quartiles) during pregnancy and ear infection, prolonged cough, pneumonia and rhinitis at 1 and 2 years of age. The risk of ear infection in the first year of life was associated with exposure to PM_2.5_ in the first trimester with adjusted OR (95% CI) = 7.64 (1.18–49.37), 11.37 (1.47–87.97) and 8.26 (1.13–60.29), respectively, for Q2–4, and similarly in the second year with adjusted OR (95% CI) = 3.28 (1.00–10.73) and 4.15 (1.05–16.36) for Q2 and 3. The association between exposures to air pollutants were significant only for the highest quartile (Q4) of PM_2.5_ and PSI in the first trimester for prolonged cough at 1 year old. As the incidence of pneumonia was low (1.8%), no significant associations were found. On the contrary, despite a high incidence of rhinitis (44.5%), we did not find statistically significant associations with air pollution.

## 4. Discussion

Our prospective birth cohort study showed that maternal exposure to ambient air pollutants during pregnancy was associated with respiratory disease in young children. We identified trimester-specific effects with exposure to higher quartiles of PM_2.5_ and PSI in the first trimester increasing the episodes of wheezing, while exposure to air pollution in the second trimester was positively associated with episodes of bronchiolitis/bronchitis. In addition, we found evidence suggestive that children of overweight or obese mothers had a higher likelihood of developing more episodes of wheezing and bronchiolitis. These associations remained significant after adjusting for potential confounders, including pollutants in other trimesters and postnatal exposure.

Ear infections in 1- and 2-year-old children were associated with PM_2.5_ exposure in the first trimester. An increased risk of prolonged cough was similarly seen in children whose mothers were exposed to the highest quartile of PM_2.5_ and PSI in the first trimester, although this effect was observed only in the first year of life. These positive associations were found although PM_2.5_ was well within the mean concentration guideline and PSI was generally within “good” ranges as stipulated by the air-quality guidelines established by the WHO [27].

Our findings are in line with several other studies [28,29,30,31]. A Mexican study group reported that children born to mothers who were exposed to both higher PM_2.5_ and stress during the first trimester had higher risk of current wheeze (relative risk (95% CI) = 1.35 (1.00–1.83)) [32]. Based on findings from a Spanish birth cohort, higher risks of lower respiratory tract infection (LRTI), wheezing and ear infection were reported in children whose mothers were exposed to NO_2_ and benzene prenatally, especially during the second trimester (for NO_2_ exposure, RR for LRTI (95% CI) 1.08 (1.02–1.15), wheezing = 1.05 (0.99–1.11), ear infection = 1.16 (0.98–1.37); for benzene exposure, LRTI = 1.10 (1.01–1.20), wheezing = 1.02 (0.96–1.09); ear infection = 1.13 (1.00–1.27)) [33]. The INMA study, however, did not find statistically significant associations between maternal NO_2_ exposure during pregnancy and common respiratory diseases among young children such as LRTI, wheezing as well as persistent cough during the first year of life [34].

Associations between maternal pre-pregnancy BMI and childhood respiratory conditions have been examined previously [20,21]. A UK-based birth cohort found that maternal pre-pregnancy BMI was associated with offspring wheeze (RR (95% CI) = 1.09 (1.05–1.13)), prolonged cough (RR (95% CI) = 1.09 (1.03–1.14)) and LRTI (RR (95% CI) = 1.13 (1.07–1.20)) [18]. Based on findings from a Dutch birth cohort positive correlations between maternal BMI and offspring’s respiratory health were reported, with every 1 kg·m^−2^ increase in maternal BMI, 2.3% more wheezing days (95% CI 1.01–1.04%) was observed in the first year of life, and 3.3% more healthcare visits for wheezing illnesses (95% CI 1.00–1.07%) until 5 years of age [19]. A large cohort study reported that maternal pre-pregnancy BMI ≥ 35 kg/m^2^ was associated with children’s current severe asthma (adjusted OR, 1.87; 95% CI, 0.95–3.68) and late-onset wheezing (adjusted OR, 1.87; 95% CI, 1.28–2.73) at 7 years of age [22]. However, to the best of our knowledge, our study is the first study to evaluate the effect modification of maternal pre-pregnancy BMI on the association of air pollution with child respiratory health.

Although Singapore has experienced recurrent acute haze crisis due to forest fire from neighboring countries, only a handful of studies have evaluated the relationship between air pollution exposure and health outcomes in Singaporeans. Chew et al. reported a positive correlation between air pollutant levels in 1990-4 and emergency room visits for acute asthma in young children [23]. Another study investigating the impact of the 1997 haze found an increase in outpatient clinic attendance for upper respiratory tract illness, asthma and rhinitis when PM10 levels increased 3-fold from 50 µg/m^3^ to 150 µg/m^3^ [25]. Despite this, no previous studies have investigated the effect of prenatal air pollution exposure on offspring respiratory health in Singapore.

We acknowledge several limitations in this study. Firstly, despite the possible spatial and temporal variations of outdoor air pollutant, the exposure was not categorized based on each participant’s home or work addresses but was averaged across the country. This method was employed as the National Environment Agency (NEA) of Singapore specified that due to Singapore’s small geographical size of 719 km^2^, the variation of air quality across the island is not significant [35]. Secondly, PSI was used instead of individual pollutants such as SO_2_, NO_2_, CO and O_3_ to represent the ambient air pollution. We note that it is important to investigate the role of these individual pollutants as their effects have been observed in other studies [10,11,32]. There are few studies that used PSI as an air quality indicator. A recent study in Taiwan found that PSI modestly increased the occurrence of atopic dermatitis (OR (95% CI) = 1.02 (1.01–1.03)) in adults [36]. Another study in Taiwan showed that change in PSI did not affect asthma hospitalization of children within a two-year period [37]. In addition, meteorological parameters, including temperature and relative humidity, can influence the concentration of air pollutants in ambient air [38]. However, there is no significant seasonal variability in tropical Singapore which is situated near the equator. Singapore is typically hot with high humidity, and does not show large variation in temperature and relative humidity throughout the year [39]. Finally, parental self-reported interviewer-administered questionnaires were used to record the child’s health status. Some recall bias and under-reporting could potentially have originated through this. Nevertheless, a recently published study reported good agreement between parental reports and doctor diagnosis for questionnaire-based wheezing data in children below 3 years of age [40]. As we follow this cohort, we will plan to evaluate the impact of air pollution on other health outcomes such as asthma at a later age. There are several strengths in this study. As this is a prospective birth cohort study, exposure is measured before the onset of disease and we could assign the air pollutant level to periods of the pregnancy to examine trimester specific effects. Important covariates, such as maternal atopy, were available for adjustment. We are aware of no similar studies previously conducted in a Singapore population.

## 5. Conclusions

In conclusion, our prospective birth cohort study showed that maternal exposure to ambient air pollution during pregnancy was associated with the incidence of respiratory health issues in the offspring. We found trimester-specific effects with effect modification by maternal pre-pregnancy BMI. Our findings support the DOHaD hypothesis that early risk factors causally influence predisposition to health and disease, although the underlying mechanism requires further investigation. This work highlights the need for more effective measures of air pollution control and prenatal care to minimize exposure to air pollution during pregnancy. More work in this birth cohort is needed to determine whether exposure to air pollution during fetal life is associated with the development of other diseases, including asthma, at later ages.

## Figures and Tables

**Table 1:** Respiratory health questions.

S/N	Question	Respiratory Health Condition
1	Has your child ever wheezed? If yes, specify number of wheezing episodes.	Wheezing
2	Has your child ever been diagnosed with bronchiolitis/bronchitis? If yes, specify number of episodes.	Bronchiolitis/bronchitis
3	Has your child ever been diagnosed with pneumonia?	Pneumonia
4	Has your child had a cough for a long period of time (e.g., 1 month)?	Prolonged cough
5	Has your child had running nose, blocked or congested nose, snoring or noisy breathing during sleep or when awake that has lasted for 2 or more weeks duration?	Rhinitis
6	Has your child ever been diagnosed by a doctor as having an ear infection?	Ear infection

**Table 2:** Demographics and incidence of respiratory conditions * of study participants.

Characteristics	Total (*n* = 953)	Children without Respiratory Conditions (*n* = 377)	Children with Respiratory Conditions (*n* = 576)	*p* Value
**Maternal**				
Age at delivery, years (mean, standard deviation (SD))	31.5 (5.1)	31.7 (5.2)	31.4 (5.1)	0.315
Ethnicity (%)				0.062
Chinese	57.5	61.7	54.7	
Malay	24.5	20.7	26.9	
Indian	18.1	17.6	18.4	
Educational attainment (%)				0.760
University	35.4	34.3	36.1	
Post-secondary/Pre-university	34.8	34.6	35	
No education/Primary/Secondary	29.8	31.1	28.9	
Multipara (%)	55.4	54.9	55.7	0.803
Maternal smoking during pregnancy and environmental tobacco smoke (ETS) ^#^ (%)				0.087
1. No	54.0	57.9	51.5	
2. Low	30.2	29.0	31.0	
3. Mild	12.0	9.0	13.9	
4. High ETS or/and active smoker	3.8	4.1	3.6	
History of maternal atopy (%)	37.8	32.3	41.3	0.005
Pre-pregnancy BMI (mean, SD)	22.7 (4.4)	22.5 (4.1)	22.9 (4.5)	0.179
**Child**				
Gestational age, weeks (mean, SD)	38.3 (1.7)	38.4 (1.6)	38.2 (1.8)	0.075
Preterm (<37 weeks) (%)	7.9	6.9	8.5	0.367
Gender-boy (%)	52.8	49.1	55.2	0.064
Birth weight, grams (mean, SD)	3090 (485)	3104 (467)	3081 (497)	0.465
Passive smoke exposure to 2 years (%)	38.5	37.1	39.4	0.462
Respiratory conditions * (%)				
Wheezing	29.2	0	29.2	
Bronchiolitis/bronchitis	13.7	0	13.7	
Pneumonia	1.8	0	1.8	
Prolonged cough	15.1	0	15.1	
Rhinitis	44.5	0	44.5	
Ear infection	5.1	0	5.1	

# never smoke and never exposed to ETS and cotinine level lower than detection limit (LOD); self-reported ETS exposure and cotinine; level < LOD; cotinine levels 0.17–13.99+ currently not smoking; cotinine levels > 14 or self-reported smoker.

**Table 3:** Statistics of exposure to air pollutants during pregnancy.

	Pregnancy			
**PM_2.5_ (μg/m^3^)**	**Entire Pregnancy**	**1st Trimester**	**2nd Trimester**	**3rd Trimester**
mean	17.92	18.21	18.24	17.17
SD	1.31	2.97	2.68	2.39
25th percentile	16.84	16.18	16.25	15.12
50th percentile	17.59	17.39	17.79	16.66
75th percentile	19.07	19.19	19.88	18.95
**PSI**				
mean	35.15	35.36	35.60	34.36
SD	1.01	3.06	2.85	2.86
25th percentile	34.25	33.30	33.51	31.79
50th percentile	35.24	35.19	35.81	34.14
75th percentile	36.09	36.92	37.73	36.43

PSI—pollutant standards index. PM_2.5_ (μg/m^3^)—particulate matter less than 2.5 mm in aerodynamic diameter. SD—standard deviation.

**Table 4:** Adjusted incidence rate ratio (IRR) and 95% confidence intervals (CI) for the association of ambient air pollution (PM_2.5_ and PSI in quartiles *) during pregnancy by trimester and episodes of wheezing by 2 years old.

					Stratified by Pre-Pregnancy BMI ≥23			
		Trimester				Trimester		
Episodes of Wheezing	Entire Pregnancy	First	Second	Third	Entire Pregnancy	First	Second	Third
**Model 1**								
PM_2.5_ Q2	1.07 (0.85–1.34)	1.53 (1.22–1.93) *	0.73 (0.59–0.91) *	1.41 (1.12–1.77) *	0.89 (0.64–1.25)	1.75 (1.24–2.48) *	0.90 (0.65–1.25)	1.43 (1.02–1.99) *
Q3	1.36 (1.10–1.69) *	1.50 (1.20–1.87) *	0.85 (0.70–1.04)	1.32 (1.04–1.67) *	1.08 (0.78–1.48)	1.54 (1.10–2.17) *	1.29 (0.94–1.76)	1.42 (1.00–2.02) *
Q4	1.32 (1.06–1.65) *	1.57 (1.26–1.97) *	0.70 (0.56–0.87) *	1.62 (1.29–2.02) *	1.33 (0.96–1.85)	1.81 (1.27–2.56) *	0.72 (0.52–1.01)	1.39 (1.00–1.94) *
PSI Q2	1.21 (0.96–1.51)	1.25 (1.00–1.56) *	0.97 (0.79–1.19)	1.27 (1.01–1.60) *	0.96 (0.68–1.34)	1.21 (0.88–1.66)	1.26 (0.93–1.71)	1.46 (1.03–2.05) *
Q3	1.26 (1.00–1.58) *	1.13 (0.91–1.41)	0.80 (0.64–1.00)	1.22 (0.97–1.54)	1.13 (0.81–1.59)	0.81 (0.57–1.15)	0.87 (0.61–1.24)	1.44 (1.02–2.04) *
Q4	1.67 (1.34–2.07) *	1.30 (1.04–1.62) *	0.80 (0.65–1.00)	1.54 (1.25–1.91) *	1.39 (1.02–1.89) *	1.25 (0.90–1.74)	0.94 (0.68–1.31)	1.40 (1.02–1.92) *
**Model 2 (multi-trimester)**								
PM_2.5_ Q2	(-)	1.55 (1.19–2.01) *	0.82 (0.61–1.10)	1.22 (0.95–1.56)	(-)	1.82 (1.24–2.67) *	1.04 (0.67–1.61)	1.24 (0.87–1.78)
Q3	(-)	1.33 (0.98–1.82)	1.29 (0.92–1.80)	1.28 (0.92–1.77)	(-)	1.70 (1.06–2.72) *	2.42 (1.43–4.09) *	1.50 (0.92–2.44)
Q4	(-)	1.56 (1.18–2.06) *	1.05 (0.77–1.43)	1.56 (1.07–2.28) *	(-)	2.07 (1.37–3.12) *	1.33 (0.81–2.19)	1.76 (0.98–3.18)
PSI Q2	(-)	1.31 (1.05–1.65) *	1.14 (0.88–1.47)	1.33 (1.03–1.71) *	(-)	1.29 (0.93–1.80)	1.70 (1.12–2.57) *	1.51 (1.03–2.22) *
Q3	(-)	0.92 (0.71–1.18)	1.07 (0.72–1.57)	1.31 (0.96–1.80)	(-)	0.65 (0.44–0.96)	1.18 (0.64–2.19)	1.62 (0.98–2.67)
Q4	(-)	1.23 (0.92–1.63)	1.14 (0.78–1.66)	1.84 (1.31–2.59) *	(-)	1.32 (0.84–2.05)	1.47 (0.81–2.67)	1.97 (1.17–3.32) *
**Model 3 (+postnatal)**								
PM_2.5_ Q2	1.50 (1.11–2.03) *	1.49 (1.13–1.96) *	0.76 (0.56–1.03)	1.29 (1.00–1.67)	1.13 (0.73–1.75)	1.85 (1.23–2.78) *	1.02 (0.65–1.59)	1.27 (0.86–1.89)
Q3	1.56 (1.17–2.08) *	1.29 (0.94–1.77)	1.22 (0.86–1.74)	1.40 (0.96–2.05)	1.15 (0.76–1.75)	1.76 (1.08–2.85) *	2.38 (1.34–4.20) *	1.52 (0.86–2.69)
Q4	1.38 (0.96–1.99)	1.27 (0.88–1.83)	1.14 (0.80–1.62)	1.60 (1.08–2.38) *	1.69 (0.99–2.91)	1.90 (1.10–3.27) *	1.45 (0.80–2.61)	1.84 (0.99–3.41)
PSI Q2	1.14 (0.87–1.49)	1.47 (1.14–1.90) *	1.08 (0.82–1.43)	1.05 (0.78–1.39)	0.70 (0.46–1.08)	1.30 (0.90–1.87)	1.48 (0.94–2.33)	1.20 (0.77–1.85)
Q3	1.37 (1.07–1.76) *	1.03 (0.78–1.37)	0.97 (0.64–1.47)	1.08 (0.75–1.54)	1.13 (0.78–1.64)	0.56 (0.37–0.86) *	0.98 (0.51–1.88)	1.45 (0.83–2.54)
Q4	1.87 (1.49–2.34) *	1.68 (1.20–2.34) *	1.31 (0.84–2.06)	1.42 (0.96–2.09)	1.45 (1.04–2.02) *	1.83 (1.12–2.98) *	1.48 (0.94–2.33)	1.82 (1.02–3.25) *

* Reference to first quartile (Q). Adjusted with maternal age at delivery, ethnicity, education level, parity, maternal atopy, maternal smoking during pregnancy and ETS, child’s gender, gestation age, and birthweight.

**Table 5:** Adjusted IRR and 95% CI for the association of ambient air pollution (PM_2.5_ and PSI in quartiles *) during pregnancy and episodes of bronchiolitis/bronchitis by 2 years old.

					Stratified by Pre-Pregnancy BMI ≥ 23			
		Trimester				Trimester		
Episodes of Bronchiolitis/Bronchitis	Entire Pregnancy	First	Second	Third	Entire Pregnancy	First	Second	Third
**Model 1**								
PM_2.5_ Q2	0.76 (0.51–1.14)	1.26 (0.89–1.78)	1.19 (0.80–1.75)	1.35 (0.96–1.91)	0.81 (0.44–1.49)	1.33 (0.75–2.37)	2.44 (1.25–4.77) *	1.36 (0.81–2.28)
Q3	1.13 (0.78–1.63)	1.04 (0.73–1.47)	1.65 (1.16–2.35) *	0.84 (0.57–1.25)	1.00 (0.56–1.79)	0.73 (0.39–1.36)	3.39 (1.77–6.47) *	0.77 (0.43–1.40)
Q4	1.28 (0.88–1.84)	1.13 (0.80–1.61)	1.26 (0.87–1.83)	1.25 (0.88–1.78)	1.63 (0.92–2.89)	1.66 (0.95–2.90)	2.63 (1.36–5.05) *	0.94 (0.55–1.61)
PSI Q2	0.47 (0.31–0.72) *	0.70 (0.46–1.05)	1.33 (0.91–1.96)	1.75 (1.20–2.54) *	0.55 (0.30–1.03)	0.98 (0.55–1.74)	2.03 (1.12–3.67) *	1.34 (0.76–2.38)
Q3	0.80 (0.55–1.16)	1.10 (0.78–1.55)	1.52 (1.03–2.24) *	0.91 (0.59–1.41)	0.73 (0.40–1.34)	1.08 (0.62–1.89)	1.30 (0.66–2.56)	1.24 (0.68–2.25)
Q4	1.25 (0.90–1.75)	1.04 (0.72–1.50)	1.06 (0.71–1.60)	1.38 (0.95–2.01)	1.24 (0.76–2.03)	1.15 (0.64–2.08)	1.57 (0.85–2.89)	0.89 (0.52–1.55)
**Model 2 (multi-trimester)**								
PM_2.5_ Q2	(-)	1.13 (0.73–1.75)	2.29 (1.34–3.89) *	1.02 (0.69–1.52)	(-)	1.14 (0.60–2.16)	5.19 (2.20–12.24) *	1.17 (0.65–2.11)
Q3	(-)	1.08 (0.64–1.81)	3.71 (2.05–6.70) *	0.69 (0.38–1.23)	(-)	1.01 (0.42–2.42)	11.06 (4.12–29.65) *	0.62 (0.24–1.59)
Q4	(-)	1.83 (1.18–2.84) *	2.37 (1.38–4.09) *	2.47 (1.33– 4.55) *	(-)	2.76 (1.45–5.26) *	4.84 (1.90–12.36) *	3.40 (1.25–9.20) *
PSI Q2	(-)	0.72 (0.47–1.10)	1.82 (1.16–2.85) *	1.67 (1.07–2.59) *	(-)	0.92 (0.50–1.70)	2.57 (1.22–5.41) *	1.62 (0.79–3.29)
Q3	(-)	1.31 (0.88–1.94)	2.20 (1.09–4.45) *	0.92 (0.50–1.70)	(-)	1.32 (0.72–2.44)	1.50 (0.47–4.74)	1.09 (0.43–2.77)
Q4	(-)	1.74 (1.11–2.71) *	1.94 (0.97–3.901)	1.54 (0.81–2.90)	(-)	1.93 (0.93–4.02)	2.07 (0.68–6.26)	0.89 (0.33–2.39)
**Model 3 (+postnatal)**								
PM_2.5_ Q2	1.14 (0.67–1.94)	1.36 (0.85–2.15)	1.90 (1.08–3.35) *	0.91 (0.60–1.39)	0.80 (0.36–1.78)	2.07 (0.97–4.40)	5.21 (2.13–12.75) *	0.74 (0.37–1.49)
Q3	1.44 (0.88–2.37)	1.02 (0.59–1.76)	2.43 (1.27–4.63) *	0.47 (0.23–0.94) *	1.10 (0.53–2.25)	1.08 (0.43–2.75)	6.05 (2.04–17.92) *	0.21 (0.06–0.67) *
Q4	1.30 (0.70–2.39)	0.75 (0.41–1.37)	1.74 (0.93–3.25)	2.36 (1.22–4.56) *	0.92 (0.37–2.29)	1.13 (0.44–2.90)	2.32 (0.80–6.72)	2.91 (1.02– 8.29) *
PSI Q2	0.46 (0.28–0.78) *	0.92 (0.58–1.47)	1.54 (0.96–2.48)	1.56 (0.96–2.56)	0.44 (0.20–0.98) *	1.24 (0.63–2.40)	2.44 (1.11–5.37) *	2.00 (0.94–4.27)
Q3	0.95 (0.63–1.44)	1.67 (1.07–2.60) *	2.18 (1.05–4.56) *	0.92 (0.48–1.78)	0.72 (0.36–1.42)	1.90 (0.96–3.75)	2.29 (0.65–8.05)	1.53 (0.55–4.27)
Q4	1.29 (0.91–1.84)	1.80 (1.05–3.10) *	2.32 (1.06–5.08) *	1.51 (0.75–3.02)	1.00 (0.57–1.73)	1.61 (0.69–3.72)	5.47 (1.42–20.99) *	1.06 (0.37–2.99)

* Reference to first quartile (Q). Adjusted with maternal age at delivery, ethnicity, education level, parity, maternal atopy, maternal smoking during pregnancy and ETS, child’s gender, gestation age, and birthweight.

**Table 6:** Adjusted odds ratio (OR) and 95% CI for the association of ambient air pollution (PM_2.5_ and PSI in quartiles *) during pregnancy and childhood respiratory health at 1 and 2 years old.

		Trimester		
	Entire Pregnancy	First	Second	Third
**Ear Infection**				
***1 year***				
PM_2.5_ Q2	2.20 (0.55–8.75)	7.64 (1.18–49.37) *	0.42 (0.06–2.92)	1.03 (0.28–3.82)
Q3	2.92 (0.85–10.05)	11.37 (1.47–87.97) *	0.28 (0.03–2.49)	0.37 (0.04–2.93)
Q4	6.40 (0.95–43.18)	8.26 (1.13–60.29) *	1.07 (0.13–8.77)	0.31 (0.03–2.92)
PSI Q2	3.45 (0.69–17.26)	1.39 (0.31–6.15)	1.60 (0.35–7.26)	1.40 (0.38–5.06)
Q3	3.15 (0.62–16.04)	1.75 (0.37–8.27)	0.90 (0.09–8.54)	0.33 (0.04–2.72)
Q4	4.25 (0.84–21.35)	2.40 (0.44–13.11)	1.09 (0.12–9.55)	1.34 (0.20–8.94)
***2 years***				
PM_2.5_ Q2	1.96 (0.53–7.15)	3.28 (1.00–10.73) *	0.48 (0.12–1.89)	0.67 (0.24–1.82)
Q3	1.77 (0.51–6.11)	4.15 (1.05–16.36) *	0.45 (0.09–2.19)	0.72 (0.16–3.17)
Q4	3.64 (0.71–18.69)	4.28 (0.92–19.87)	1.00 (0.21–4.66)	0.25 (0.04–1.47)
PSI Q2	2.50 (0.83–7.45)	1.53 (0.51–4.57)	1.54 (0.46–5.09)	0.95 (0.31–2.90)
Q3	2.01 (0.70–5.71)	1.69 (0.51–5.55)	1.42 (0.26–7.78)	0.44 (0.09–2.14)
Q4	2.07 (0.71–6.03)	3.42 (0.86–13.53)	1.56 (0.25–9.57)	0.64 (0.12–3.50)
**Prolonged cough**				
***1 year***				
PM_2.5_ Q2	1.66 (0.74–3.70)	1.94 (0.81–4.65)	1.04 (0.42–2.59)	0.88 (0.40–1.95)
Q3	1.29 (0.60–2.78)	1.15 (0.40–3.32)	1.64 (0.58–4.66)	0.76 (0.23–2.48)
Q4	2.56 (0.89–7.34)	3.03 (1.07–8.52) *	1.08 (0.36–3.23)	1.64 (0.51–5.26)
PSI Q2	1.94 (0.89–4.25)	2.03 (0.89–4.65)	1.98 (0.85–4.60)	0.97 (0.42–2.24)
Q3	2.45 (1.13–5.30) *	1.16 (0.47–2.82)	1.25 (0.34–4.48)	0.85 (0.31–2.32)
Q4	2.07 (1.00–4.27) *	3.67 (1.53–8.79) *	1.66 (0.47–5.88)	1.57 (0.54–4.53)
***2 years***				
PM_2.5_ Q2	0.95 (0.47–1.91)	1.06 (0.52–2.15)	1.06 (0.49–2.28)	0.79 (0.42–1.48)
Q3	1.20 (0.62–2.34)	1.34 (0.60–2.96)	1.38 (0.55–3.44)	0.83 (0.32–2.11)
Q4	1.66 (0.71–3.87)	1.52 (0.64–3.57)	1.27 (0.52–3.09)	1.32 (0.50–3.45)
PSI Q2	0.92 (0.47–1.77)	1.25 (0.67–2.34)	1.42 (0.72–2.81)	1.21 (0.59–2.47)
Q3	1.37 (0.77–2.44)	0.88 (0.45–1.74)	1.20 (0.43–3.31)	1.03 (0.44–2.40)
Q4	1.45 (0.84–2.48)	1.86 (0.86–3.99)	1.14 (0.38–3.40)	1.74 (0.70–4.33)
**Pneumonia**				
***1 year***				
PM_2.5_ Q2	0.36 (0.03–4.42)	9.75 (0.585–162.77)	1.05 (0.05–19.62)	0
Q3	1.47 (0.20–10.50)	10.45 (0.39–278.03)	3.31 (0.13–80.25)	0.77 (0.03–19.29)
Q4	0.84 (0.05–13.82)	5.98 (0.20–178.81)	1.52 (0.05–41.76)	0.33 (0.009–11.92)
PSI Q2	0.52 (0.04–6.16)	0.76 (0.08–6.78)	2.22 (0.11–42.16)	0.15 (0.008–2.93)
Q3	1.21 (0.17–8.29)	0.24 (0.02–2.89)	0.19 (0.003–13.22)	0.19 (0.011 3.494
Q4	0.97 (0.12–7.64)	0.19 (0.008–4.64)	0.41 (0.008–21.61)	0.58 (0.05–6.33)
***2 years***				
PM_2.5_ Q2	1.68 (0.20–14.06)	0.87 (0.09–7.95)	1.26 (0.17–9.36)	1.08 (0.15–7.65)
Q3	3.71 (0.53–26.00)	1.26 (0.14–11.13)	1.57 (0.11–21.02)	7.51 (0.69–81.35)
Q4	3.93 (0.45–34.41)	3.67 (0.37–36.28)	5.30 (0.53–52.98)	6.00 (0.41–87.60)
PSI Q2	0.48 (0.04–4.78)	0.88 (0.13–5.79)	1.25 (0.28–5.55)	1.14 (0.14–9.22)
Q3	2.68 (0.58–12.45)	1.39 (0.21–9.19)	0.35 (0.03–3.90)	0.94 (0.11–7.91)
Q4	2.25 (0.51–9.84)	0.44 (0.05–3.85)	0.12 (0.007–2.38)	1.76 (0.19–16.36)
**Rhinitis**				
***1 year***				
PM_2.5_ Q2	1.06 (0.69–1.65)	1.10 (0.66–1.83)	0.88 (0.49–1.57)	1.25 (0.78–1.99)
Q3	0.84 (0.55–1.29)	0.81 (0.44–1.49)	1.16 (0.61–2.22)	1.16 (0.57–2.35)
Q4	1.25 (0.68–2.29)	0.82 (0.43–1.57)	1.48 (0.75–2.90)	1.65 (0.79–3.44)
PSI Q2	0.94 (0.62–1.43)	0.93 (0.60–1.44)	0.80 (0.47–1.37)	0.91 (0.56–1.47)
Q3	1.11 (0.73–1.68)	0.85 (0.52–1.38)	1.11 (0.51–2.40)	1.15 (0.64–2.06)
Q4	1.28 (0.85–1.93)	1.14 (0.67–1.96)	1.38 (0.64–2.99)	1.00 (0.52–1.92)
***2 years***				
PM_2.5_ Q2	0.98 (0.61–1.56)	1.14 (0.70–1.87)	0.68 (0.39–1.20)	1.21 (0.78–1.89)
Q3	0.90 (0.56–1.43)	0.78 (0.43–1.40)	0.82 (0.42–1.58)	0.92 (0.47–1.82)
Q4	0.93 (0.50–1.74)	0.86 (0.45–1.64)	0.92 (0.48–1.76)	1.29 (0.63–2.64)
PSI Q2	0.90 (0.57–1.42)	0.96 (0.62–1.47)	0.82 (0.49–1.37)	1.07 (0.65–1.76)
Q3	1.03 (0.68–1.56)	0.85 (0.53–1.37)	0.96 (0.46–2.00)	1.05 (0.57–1.92)
Q4	1.12 (0.75–1.67)	1.13 (0.63–2.00)	0.85 (0.38–1.89)	1.07 (0.54–2.09)

* Reference to first quartile (Q). Adjusted with maternal age at delivery, ethnicity, education level, parity, maternal atopy, maternal smoking during pregnancy and ETS, child’s gender, gestation age, and birthweight, multi-trimester pollutant, postnatal ETS and air pollution.
